# Decreased Expression of CD69 on T Cells in Tuberculosis Infection Resisters

**DOI:** 10.3389/fmicb.2020.01901

**Published:** 2020-08-07

**Authors:** Zhen-Yan Chen, Lei Wang, Ling Gu, Rong Qu, Douglas B. Lowrie, Zhidong Hu, Wei Sha, Xiao-Yong Fan

**Affiliations:** ^1^Shanghai Public Health Clinical Center, Key Laboratory of Medical Molecular Virology of MOE/MOH, Fudan University, Shanghai, China; ^2^Shanghai Pulmonary Hospital, Tongji University, Shanghai, China; ^3^School of Laboratory Medicine and Life Science, Wenzhou Medical University, Wenzhou, China; ^4^TB Center, Shanghai Emerging and Re-emerging Institute, Shanghai, China

**Keywords:** tuberculosis, CD69, T-cell, latent infection, close contacts, resister

## Abstract

**Background:**

CD69 is a biomarker of T-cell activation status, but its activation status in human *Mycobacterium tuberculosis* (*Mtb*) infection remains elusive.

**Methods:**

A set of cohorts of patients with different tuberculosis (TB) infection status including active TB patients (ATB), latent tuberculous infection patients (LTBI) and close contacts (CCs) of ATB was designed, and the expression profiles of CD69 and several T-cell markers were determined on *Mtb* antigen-stimulated T cells by flow cytometry.

**Results:**

The frequencies of CD4^+^ and CD8^+^ T cells were both comparable among *Mtb*-infected individuals including ATB and LTBI, which guaranteed the consistency of the background level. A t-Distributed Stochastic Neighbor Embedding (tSNE) analysis on a panel of six phenotypic markers showed a unique color map axis gated on T cells in the CCs group compared with ATB and LTBI populations. By further gating on cells positive for each individual marker and then overlaying those events on top of the tSNE plots, their distribution suggested that some markers were expressed differently in the CCs group. Further analysis showed that the expression levels of CD69 on both CD4^+^ and CD8^+^ T cells were significantly lower in the CCs group, especially in interferon-γ-responding T cells.

**Conclusion:**

Our findings suggest that the T-cell activation status of CD69 is associated with *Mtb* infection and may have the potential to distinguish LTBI from those populations who have been exposed continuously to *Mtb* but have not become infected.

## Introduction

Tuberculosis (TB) is the leading cause of death due to a single infectious disease in the world, there were an estimated 10 million new TB cases and 1.5 million deaths in the year of 2018 (WHO, 2019). Host-directed therapy is a promising strategy for TB treatment but it has not yielded persuasive results due to our incomplete understanding of immunological mechanism against this disease.

About a quarter of the world’s population has latent TB infection (LTBI), and their lifetime risk of developing TB disease is around 5%∼15% (WHO, 2019). *Mycobacterium tuberculosis* (*Mtb*) infection is defined by standard clinical tests including tuberculin skin tests (TST) and interferon (IFN)-γ release assays (IGRAs). Recently, it was reported that nine months of isoniazid or four months of rifampin treatment can prevent the development of active TB disease in persons with LTBI (Samanovic and Darwin, 2016), illustrating the feasibility of sterile eradiation of *Mtb* in latent infection. However, the exact risk and timing of disease resulting from the exposure of close contacts (CCs) to active TB patients (ATB) have not been determined (Reichler et al., 2018). Recently, particular attention has been placed on the group of CCs with persistently negative TST/IGRA results despite prolonged exposure, a group termed “resisters” (Simmons et al., 2018; Kaipilyawar and Salgame, 2019). Identification of differential epidemiological and immunological characteristics of the CCs that are either “resisters” or LTBI will provide greater biological insights to prevent or clear early TB infections and develop novel diagnostic methods.

T cells, especially Th1 CD4^+^ T cells, are a crucial part of anti-TB immunity. T-cell activation and exhaustion represent the different immune status at different stages of *Mtb* infection, distinguished by the expression of specific biomarkers. Our previous work demonstrated that the expression of KLRG1, regarded as a T-cell terminal differentiation biomarker, was associated with the progression of human TB (Hu et al., 2018). In animal infection/vaccination models, the expression of several T-cell biomarkers, such as CD38 (Dintwe et al., 2013), CD69 (Andersen and Smedegaard, 2000; Kauffman et al., 2018), CTLA-4 (Kirman et al., 1999; Nandakumar et al., 2014), LAG-3 (Workman et al., 2004; Phillips et al., 2015), Tim-3 (Jayaraman et al., 2016), and PD-1 (Lazar-Molnar et al., 2010; Reiley et al., 2010), has been reported to be related to TB infection. However, association with clinical TB has not been shown for all of them, especially their expression profiles in CCs remain to be defined.

In this study, the expression profiles of six biomarkers related to TB infection were compared among ATB, LTBI and CCs populations by flow cytometry in a designed cohort. As a result, Although the frequency of T cells was identical among these groups, a t-Distributed Stochastic Neighbor Embedding (tSNE) analysis on a panel including the phenotypic markers showed a unique color map axis gated on T cells in the CCs group compared with ATB and LTBI populations. By further gating on cells positive for each marker and then overlaying those events on top of the tSNE plots, their distribution suggested that some makers were differently expressed in the CCs group. Further analysis showed that the expression levels of CD69 on both CD4^+^ and CD8^+^ T cells were significantly lower in CCs group, especially on antigen-specific T cells. Thus, our data suggest that the activation status of CD69 might be associated with TB disease progression, and showed the potential to distinguish CCs from LTBI populations.

## Materials and Methods

### Subjects

This study was approved by the Ethical Committee of Shanghai Pulmonary Hospital (approval number K18-215Z), and informed consent was obtained from all subjects. As shown in Table 1, the cohort comprised 45 ATB, 15 LTBI, 13 CCs and 17 non-TB-infected/close contacted persons (Non-TB). ATB was identified on the basis of sputum or effusion smear or polymerase chain reaction amplification positivity, confirmed by radiological findings and clinical syndromes, alongside with a final clinical diagnosis of ATB. LTBI was defined as IGRA-positive without clinical syndromes of active TB infection. CCs populations who were highly exposed to *Mtb* without infection fulfilled the following criteria: persons who had shared air space with an individual with pulmonary TB in the household or other indoor setting for > 15 hr per week or > 180 hr total during an infectious period (an infectious period was defined as the interval from 3 months before collection of the first culture-positive sputum specimen or the date of onset of cough, whichever was longer, through 2 weeks after the initiation of appropriate anti-tuberculosis treatment) and they were IGRA/TST negative without clinical syndromes of active TB infection. Non-TB subjects were defined as not *Mtb*-infected (IGRA-negative) and not having had close contact with ATB patients. All subjects were HIV/HCV-negative and were not taking immunosuppressive drugs.

**TABLE 1:** Clinical characteristics of study participants.

Groups	Patients with Active	Latent Tuberculosis	Close Contacts	Non-TB populations
	Tuberculosis (*n* = 45)	Infection (*n* = 15)	(*n* = 13)	(*n* = 17)
**Age, yr**	41.82 (14–77)	43.27 (17–73)	44.02 (33–75)	43.71 (23–76)
**Tuberculosis manifestation**				
Pulmonary	33.33	N/A	N/A	N/A
Extrapulmonary	0	N/A	N/A	N/A
Both	66.67	N/A	N/A	N/A
**Smear/culture/PCR positivity**	100	0	N/A	N/A
**Sex, %**				
Male	60	66.67	69.23	64.71
Female	40	33.33	30.77	35.29
**BCG vaccinated**	100	100	100	100
**HIV**	0	0	0	0

*Data are median values (range) or percentage of participants. N/A, not applicable; PCR, polymerase chain reaction.*

### Peripheral Blood Mononuclear Cells (PBMCs) Isolation

An equal volume of heparinized peripheral venous blood and RPMI-1640 cell culture medium (Hyclone) were mixed and slowly added onto an equal volume of lymphocyte separation solution Ficoll at room temperature (HMK). The diluted whole blood was layered on top of the lymphocyte separation solution with a clear interface. After centrifugation at 600 x *g* for 20 min, PBMCs were collected and washed two times with RPMI 1640 medium. The cells were suspended in 1 mL R10 medium (RPMI-1640 medium containing 10% (v/v) fetal bovine serum [FBS]). Viability and numbers of cells were determined using trypan blue in a counting chamber.

### T-Spot Assay

T-Spot assay uses an ELISpot (enzyme-linked immune absorbent spot) platform for diagnosing TB infection (Beijing Jinhao, China). The test was performed according to the instructions of the manufacturer (Li et al., 2017). Briefly, PBMCs were added into a 96-well plate which was pre-coated with antibodies against IFN-γ in a volume of 100 μL/well with or without ESAT-6 and CFP10 peptides added as stimulants. After incubation at 37° in a 5% CO_2_ incubator for 18∼20 h, the wells were washed and a working solution of a second antibody conjugated to biotin was added (100 μL/well). After incubation for 1 h at room temperature, the wells were washed and the enzyme conjugate working solution was added (100 μL/well) and incubated for another 1 h at room temperature. After washing again, the chromogenic substrate AEC working solution was added (100 μL/well) and incubated at room temperature for 7 min in the dark. Purified water was added to stop the reaction. The spots were counted by using an ImmunoSpot Reader (ChampSpot III, Beijing Sage Creation Science, China). The results were defined as positive if the spots in the detection hole numbered at least 6 when the spots in negative control hole were fewer than 6, or the spots in the detection well were twice as many as the spots in the negative control when the number in the negative control hole was greater than 6.

### Intracellular Cytokine Staining (ICS) and Flow Cytometric Analysis

The ICS assay was measured as described elsewhere (Hu et al., 2017). Briefly, the freshly isolated PBMCs were stimulated with peptide pools of ESAT-6 and CFP10 (5 μg/mL, synthesized by GL Biochem, China) in the presence of Brefeldin A and Monensin (both 1 μg/mL, BD Biosciences) in a round-bottom 96-well plate at 37° and 5% CO_2_ for 12 h. After centrifugation at 600 x *g* for 5 min at 4°, the cells were surface-stained with 50 μL of the appropriate antibody panel diluted in PBS buffer containing 2% FBS for 30 min at 4° in the dark, followed by washing, fixation and permeabilization with Cytofix/Cytoperm (BD Biosciences) for 20 min at 4° in the dark. The fixed cells were washed then treated with a panel of antibodies against intracellular cytokines in a final volume of 50 μL and incubated for another 30 min at 4° in the dark. Finally, the cells were washed and transferred into the flow tube and analyzed by flow cytometry (LSR Fortessa, BD Biosciences). The data were analyzed by FlowJo 10 software (Ashland, OR).

### t-Distributed Stochastic Neighbor Embedding (tSNE) Analysis

tSNE is a machine learning dimensionality-reducing algorithm that compares similarities of data points in high dimensional space and plots it in two dimensions through a way that preserves the structure of the dataset. In this study, firstly, all of the flow cytometry data from the ATB, LTBI and CCs groups were electronically concatenated into a single FCS file using FlowJo 10 software. Then the large concatenated files were down-sampled to create separate files for each group at a size of 50, 000 events/group. The equal-sized files were then gated on CD4^+^ T cells based on the antibodies of CD3 and CD4, and tSNE analysis was performed in FlowJo using iterations = 1000 and perplexity = 30. The number of iterations refers to the number of loops in the repeated execution of the program, the default number (=1000) was used in this study. Perplexity is associated with the number of nearest neighbors that are used in learning algorithms and can be regarded as a knob that sets the number of effective nearest neighbors in tSNE analysis. The most appropriate value depends on the density of the data, a relatively large value (=30) was used since a large dataset was analyzed in this study.

### Antibodies

The antibodies used in this study: CD3-PE/Dazzle594 (clone OKT3), CD38-FITC (clone 303504), CD69-Alexa Fluor 700 (clone 310922), Tim-3-Brilliant Violet 421 (clone F38-2E2) and CTLA-4-Percp-Cy5.5 (clone BN13) were from Biolegend. CD4-PE-Cy5 (clone RPA-T4), HLA-DR-APC (clone LN3), PD-1-APC-eFluor780 (clone EbioJ105) and IFN-γ-PE-Cy7 (clone 4S.B3) were from eBioscience.

### Statistical Analysis

Statistical analyses were done using GraphPad Prism 7 software (La Jolla, CA). The data had a normal distribution and homogeneity of variance. The statistical differences between groups were assessed using one-way ANOVA tests. *P* < 0.05 was considered statistically significant.

## Results

### Comparable T-Cell Frequencies Among ATB, LTBI, and CCs Groups

**FIGURE 1:**
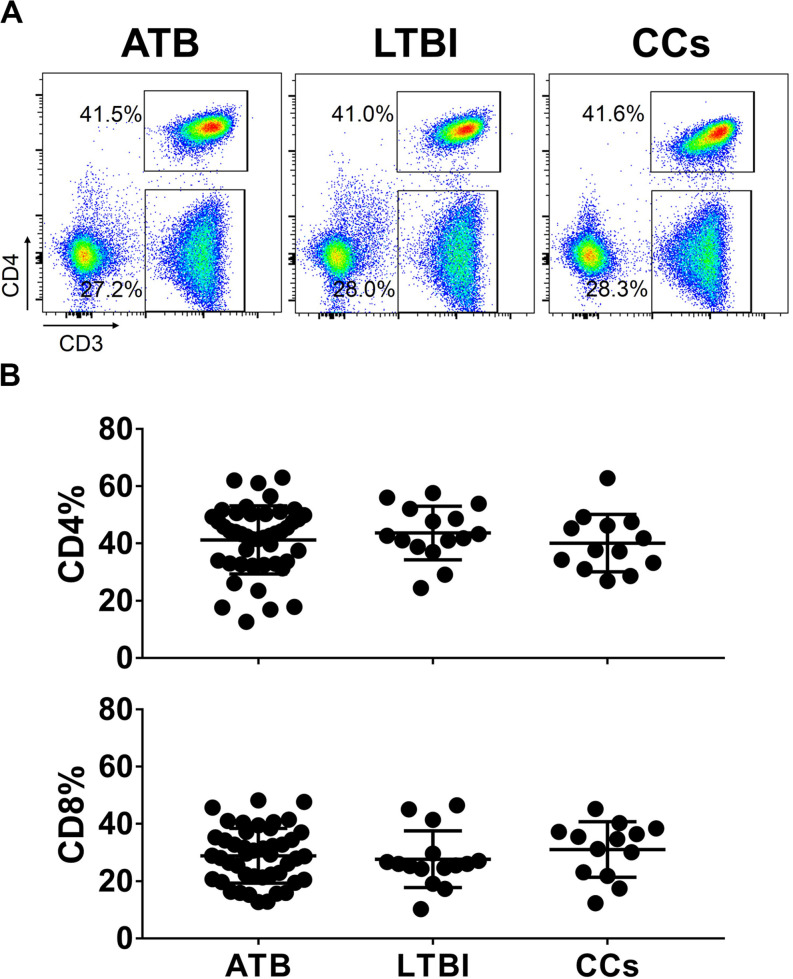
The frequencies of CD4^+^ and CD8^+^ T cell subsets among groups. The freshly isolated PBMCs were stimulated with peptide pools for 12 h, and the percentage of CD4^+^ T cells and CD8^+^ T cells were determined by flow cytometry. Representative flow cytometric dot plots are shown in **(A)**. CD4^+^ T cells were gated as CD3^+^CD4^+^ cells and CD8^+^ T cells were defined as CD3^+^CD4^–^ cells. Summarized data are shown as scatter dots in **(B)**.

To explore the role of T cells in close contacts of TB patients, we firstly compared the frequencies of CD4^+^ and CD8^+^ T cells among TB, LTBI and CCs groups (the gating strategy is shown in Supplementary Figure S1). As shown in Figures 1A,B, the percentages of CD4^+^ and CD8^+^ T cells were comparable among groups as expected.

### tSNE Analysis of a Panel Including Several Phenotypic Markers on T Cells

**FIGURE 2:**
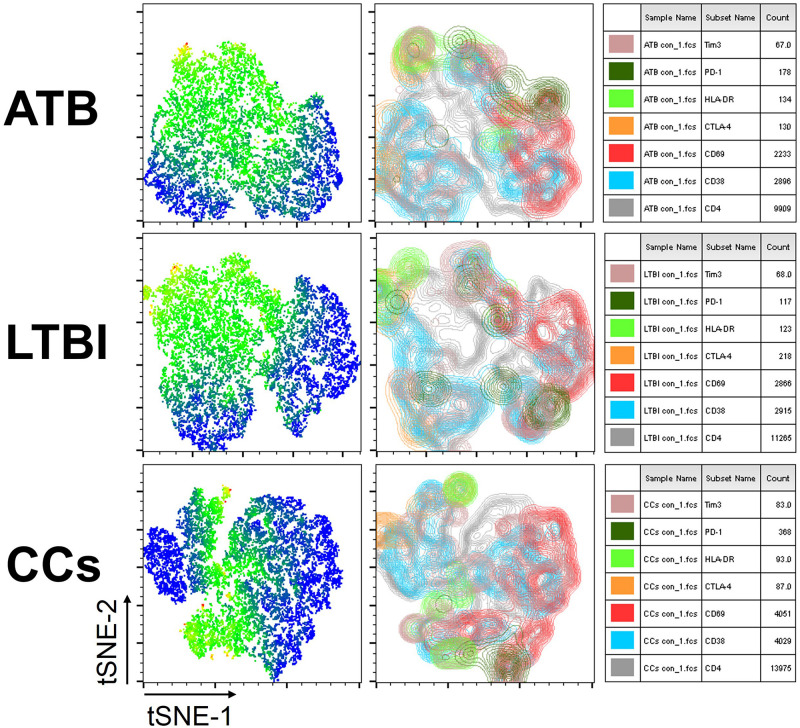
tSNE map for six biomarkers on CD4^+^ T cells. The expression levels and distribution of different biomarkers in each group demonstrated by tSNE. The heat map shows the degree of activity of different cells expressing various biomarkers, the most active is indicated by red, and the least active is indicated by blue (left panel). The classic tSNE map indicates different signs with different colors, which more intuitively shows their expression on CD4^+^ T cells (middle panel). Combining heat map and classic map, the biomarkers for each color and the numbers of lymphocytes that express each biomarker are shown (right panel).

To further delineate the profiles of T cell-mediated immune responses in the CCs group, six T-cell biomarkers that had been reported to be associated with *Mtb* infection in mouse models were used. By gating on cells positive for each marker and then overlaying those events on top of the tSNE plots, we found that the cell distribution was similar between ATB and LTBI groups in a 2D scatterplot. Interestingly, the CCs group showed a unique plot map compared with the other two groups (Figure 2). This led us to further analysis of the different expression profiles of these markers.

### CD69 Is Under-Expressed on CD4^+^ and CD8^+^ T Cells in CCs

**FIGURE 3:**
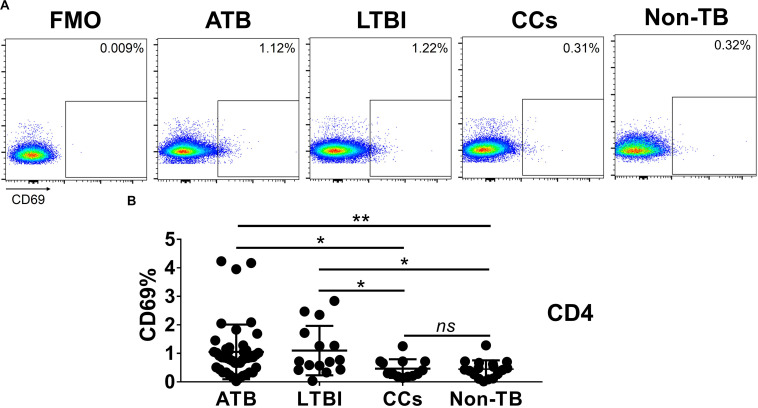
The expression levels of CD69 on CD4^+^ T cells. Representative flow cytometric plots of CD69 staining and “fluorescence minus one” (FMO) staining control on CD4^+^ T cells are shown in **(A)** and the summarized data are shown as dot scatter plots in **(B)**. Values are expressed as mean ± SD. The *P* values were calculated by one-way ANOVA and were labeled in the figures. *P < 0.05, **P < 0.01 and *ns* indicates no significant difference, when compared as indicated.

**FIGURE 4:**
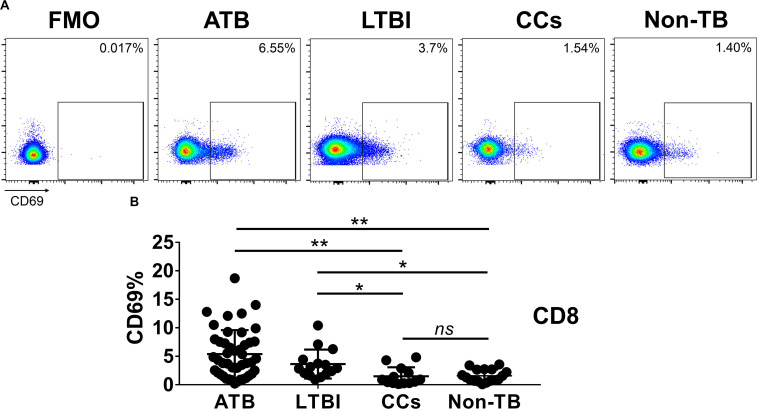
The expression levels of CD69 on CD8^+^ T cells. Representative flow cytometric plots of CD69 staining and FMO staining control on CD8^+^ T cells are shown in **(A)** and the summarized data are shown as dot scatter plots in **(B)**. Values are expressed as mean ± SD. The *P* values were calculated by one-way ANOVA and are labeled in the figures. *P < 0.05, **P < 0.01 and *ns* indicates no significant difference, when compared as indicated.

**FIGURE 5:**
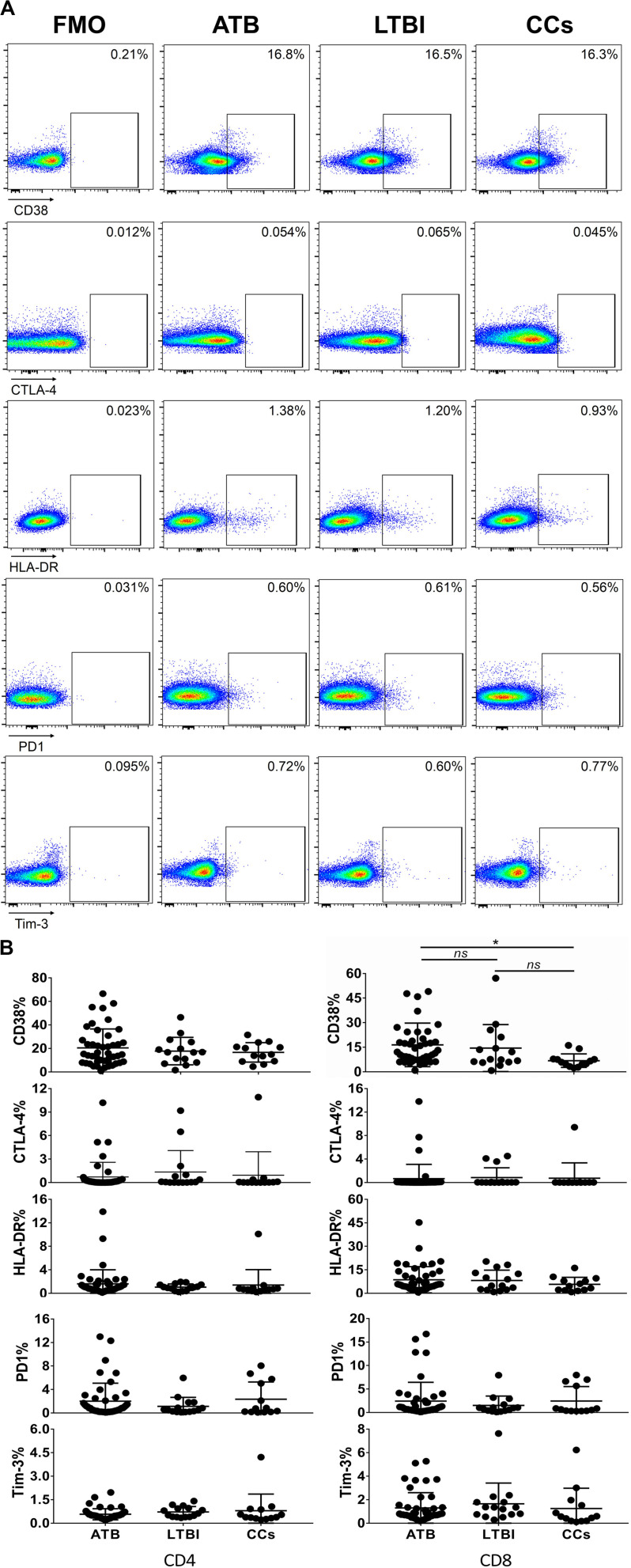
The expression profiles of CD38, CTLA-4, HLA-DR, PD1, and Tim-3 on CD4^+^ T cells and CD8^+^ T cells. Representative flow cytometric plots of CD38, CTLA-4, HLA-DR, PD1, and Tim-3 staining and FMO staining controls are shown in **(A)** and the summarized data are shown as dot scatter plots in **(B)**. Values are expressed as mean ± SD. The *P* values were calculated by one-way ANOVA and are labeled in the figures. *P < 0.05 and *ns* indicates no significant difference, when compared as indicated.

As shown in Figures 3A,B, the percentage of CD69^+^ cells among CD4^+^ T cells was significantly lower in household contacts and non-TB infected/close-contacted populations, compared with patients infected or latently infected with TB. The expression of CD69 showed no significant differences between the ATB and LTBI groups. The CCs and Non-TB groups also showed comparable CD69 expression on CD4^+^ T cells (Figure 3). The differences between groups in mean fluorescence intensity (MFI) values of CD69 on CD4^+^ T cells did not reach statistical significance (Supplementary Figure S2). Similarly, CD69 was also under-expressed on CD8^+^ T cells in CCs and Non-TB groups compared with TB infection groups (Figure 4). In contrast, the expression profiles of CD38, CTLA-4, HLA-DR, PD, and Tim-3 were comparable on both CD4^+^ T cells and CD8^+^ T cells, except that the expression of CD38 on CD8^+^ T cells was lower in CCs groups than in ATB patients (Figure 5). Thus, these data suggest that the activation status of CD69 is associated with *Mtb* infection and may have the potential to distinguish LTBI from those populations who are exposed continuously to *Mtb* but are not infected.

### The Expression of CD69 on IFN-γ-Responding T Cells

**FIGURE 6:**
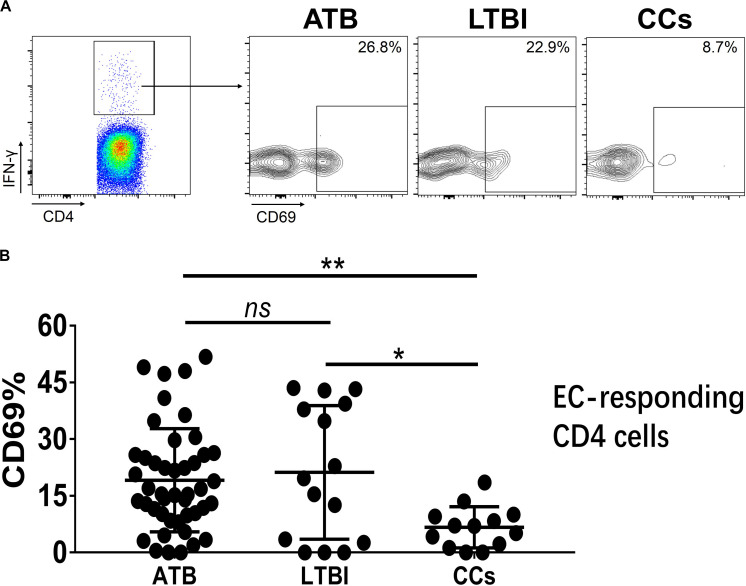
The expression levels of CD69 on IFN-γ-responding CD4^+^ T cells. Representative flow cytometric plots of IFN-γ and CD69 staining on CD4^+^ T cells are shown in **(A)** and the summarized data are shown as dot scatter plots in **(B)**. EC, ESAT-6, and CFP10 peptide pools. Values are expressed as mean ± SD. The *P* values were calculated by one-way ANOVA and are labeled in the figures. *P < 0.05, **P < 0.01 and *ns* indicates no significant difference, when compared as indicated.

**FIGURE 7:**
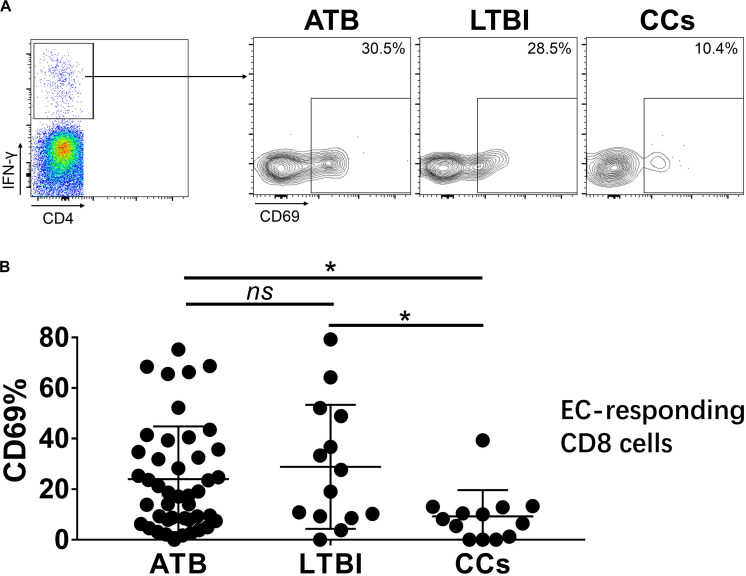
The expression levels of CD69 on IFN-γ-responding CD8^+^ T cells. Representative flow cytometric plots of IFN-γ and CD69 staining on CD8^+^ T cells are shown in **(A)** and the summarized data are shown as dot scatter plots in **(B)**. Values are expressed as mean ± SD. The *P* values were calculated by one-way ANOVA and were labeled in the figures. *P < 0.05 and *ns* indicates no significant difference, when compared as indicated.

To further characterize the association of CD69 with the status of TB infection, we tested the frequency of CD69 on *Mtb*-specific T cells, which were defined as T cells that responded to produce IFN-γ after stimulation with *Mtb* antigens ESAT-6 and CFP10. Although most CCs were IGRA-negative, they showed a slight IFN-γ secretion at a background level in ICS assay, thus facilitating our analysis. Interestingly, our data showed that the expression levels of CD69 on IFN-γ^+^ CD4^+^ T cells were significantly lower in the CCs group compared with the ATB (*P* < 0.01) and LTBI (*P* < 0.05) groups (Figure 6), which was a higher degree of significance than found with the total CD4^+^ T cells (Figure 3). In addition, the CD69^+^ frequency was also significantly lower in IFN-γ^+^ CD8^+^ T cells in CCs compared with ATB (*P* < 0.05) and LTBI (*P* < 0.05) groups (Figure 7).

Taken together, our data showed a lower expression of CD69 on T cells, especially on *Mtb* antigen-stimulated IFN-γ-responding T cells, in the CCs group compared with TB infection groups, indicating that persistent CD69 activation is associated with the impaired host defense against *Mtb* infection that culminates in disease. It was recently shown that “resisters”, defined as CCs with persistent TST/IGRA negativity, displayed enhanced antibody avidity and distinct *Mtb*-specific IgG Fc profiles instead of IFN-γ T-cell responses to ESAT-6 and CFP10 (Lu et al., 2019). Thus, our data support the contention that a CD69-expressing/IFN-γ-secreting non-T-cell host response to *Mtb* exposure might exist within the CCs population. Furthermore, lower CD69 expression in response to the *Mtb* antigen stimulation might be a clinically significant characteristic that could serve to distinguish CCs from TB-infected populations.

## Discussion

Despite significant progress over the past few decades, TB remains the world’s leading killer infectious disease (WHO, 2019), suggesting that a more thorough understanding of the immune characteristics of *Mtb* infection is needed.

Especially needed are TB-specific biomarkers as indicators to predict infection reactivation, disease prognosis or vaccine-induced immune protection, and to differentiate various TB infection stages. Measurement of differences in T-cell activation or exhaustion levels will be helpful. Previously, we demonstrated the role of T-cell surface maker KLRG1 in human CD4^+^ T-cell immunity against TB (Hu et al., 2018). Recently, the concept of “resisters”, defined as CCs who are persistently TST/IGRA negative, was described. “Resisters” were found to generate a non-IFN-γ-centric, *Mtb*-specific, CD4^+^ T-cell-mediated immune response to TB exposure, marked by high levels of up-regulated CD40L/CD154 in a clinical cohort study (Lu et al., 2019). This finding served to highlight the importance of T-cell surface marker expression in anti-TB T-cell-mediated immunity. Although a range of biomarkers was found associated with TB infection in mouse models, not all have been clinically verified and in particular, their expression profiles in CCs remain to be defined.

Our new finding, that CD69 was under-expressed in CCs group, suggested that it might be associated with *Mtb* infection. Considering that the sample size in our current cohort was not large, we also determined CD69’s expression profiles in non-*Mtb* infected/close contact (Non-TB) persons. Consistent with our expectation, the CD69’s expression was significantly lower in the Non-TB persons compared with ATB and LTBI, further supporting our conclusion that activation status of CD69 on T cells was affected by *Mtb* infection.

CD69, a type II glycoprotein, is one of the earliest cell surface antigens expressed by T cells following activation, and potentially plays an important role in the activation and differentiation of a wide variety of hematopoietic cells (Ziegler et al., 1994). Numerous viral and bacterial infection models showed increased CD69 expression on T cells (Hodge et al., 2004; Vega-Ramos et al., 2010; Ishikawa et al., 2013). CD69 is also a co-stimulatory biomarker commonly expressed on CD8^+^ T cells that bind to corresponding ligands that are expressed on DCs (Maimela et al., 2018). CD69 activation stimulates an influx of calcium ions and the activation of extracellular kinases ERK1/2, thereby facilitating CD8^+^ T cell proliferation. In addition, CD69 activation stimulates the secretion of IL-2 and IFN-γ that promotes the cytotoxic function of CD8^+^ T cells (Gonzalez-Amaro et al., 2013). CD69 is preferentially expressed on cells that activate the memory phenotype (CD45RO^+^ HLA-DR^+^), with an increase in the mucosal homing signal CCR6 and a decrease in the secondary lymphoid tissues homing signals CCR7 and CD62L. This suggests that CD69 may help lymphocytes migrate to the site of infection (Sallusto et al., 1999; Yong et al., 2017). The expression levels of CD69 on T cells fluctuated during anti-TB chemotherapy (Portales-Pérez et al., 2002) and in HIV-1/TB co-infected patients, the percentage of CD4+ T cells expressing CD69^+^ was related to TST and IGRA results (Hsieh et al., 2000) confirming an association with disease status. Because here we have demonstrated that there is increased CD69 expression on CD4^+^ and CD8^+^ T cell in ATB and LTBI we speculate that blockage of CD69 by a specific antibody might influence the function of CD4^+^ T cells. This remains to be investigated.

It was reported that primary viral infection might cause an IFN-I-dependent and systemic “partial” activation of T lymphocytes that was characterized by up-regulated expression of early activation marker CD69 and co-stimulatory molecule CD86 (Alsharifi et al., 2006). These partially activated T cells survived better and developed into effector cells more efficiently and thereby helped to eliminate virus infection in the early infection phase (Wijesundara et al., 2010). However, during chronic viral/bacterial infection and tumor formation, the ability of T cells to function and to establish memory might be impaired due to persistent/chronic antigen stimulation and then ultimately weaken the ability of the cells to confer host protection. This phenomenon was termed “T-cell exhaustion” (Blank et al., 2019). Exhausted T cells display high levels of surface markers such as PD-1, Tim-3, CTLA-4, LAG-3, and CD69 (Yi et al., 2010). Although the application of so-called “immune checkpoint blockade” therapies that target these molecules has become a major weapon in fighting cancer, a greater understanding of T-cell exhaustion is imperative to establish rational immunotherapeutic interventions (Hashimoto et al., 2018). Previously, we showed that the expression of KLRG1, a maker of terminally differentiated T cells, was significantly increased during persistent TB infection, leading to an inadequate T-cell immune response (Hu et al., 2018). With regard to CD69, it was reported that targeting CD69 enhanced the early control of virus infection which related to increased numbers of cytokine-producing T cells and NK cells in the periphery (Notario et al., 2019). The attenuation of tumor progression in CD69 knockout mice was related to the increased levels of tumor infiltrating T cells and the decreased levels of CD8 T-cell exhaustion, and anti-CD69 antibody treatment enhanced the anti-tumor activity (Mita et al., 2018). In this study, we showed that *Mtb* infection similarly led to increased levels of CD69 expression on T cells, whereas CCs population showed levels of CD69 expression similar to those in non-TB infected/close contacted population. Thus, our data suggest that persistent CD69 activation might impair host defense against *Mtb* infection.

Considering all the subjects were immunized with BCG vaccines, ESAT-6 and CFP10, which are *Mtb*-specific antigens and not secreted by BCG strains, were used throughout the study, to avoid potential confounding factors (Yang et al., 2013). Even though, BCG-specific T-cell immune memory might also be associated with TB disease status, and it was showed that BCG increased CD69’s expression on both CD4 (Hougardy et al., 2007) and natural killer cells (Marcenaro et al., 2008) in human cohort studies. Thus, it’s reasonable to speculate that BCG-stimulated CD69’s expression might also be used to define TB infection status, which worth further exploration.

It should be noted that CD8 T cells were roughly defined as CD3^+^CD4^–^ cells in this study, however, several T-cell subsets such as γδ T cells, NKT cells and mucosal-associated invariant T cells might be CD3^+^CD4^–^CD8^–^, and they are not rare in peripheral blood (D’Acquisto and Crompton, 2011). Indeed, these T-cell subsets have been shown to participate in human host defense against *Mtb* infection in patients with active TB (Behr-Perst et al., 1999; Gansert et al., 2003; Jiang et al., 2014) and a decrease of CD69 expression on TCR Vα7.2^+^ CD4^–^ T cells was reported to be associated with impaired cytotoxic functions in chronic hepatitis B virus-infected patients (Yong et al., 2017). Thus, the surface markers such as CD69 on these subsets also have the potential to differentiate between LTBI and CC groups, which is a possibility worth further evaluation.

In conclusion, this study demonstrated that an up-regulation of the T-cell phenotype that expresses CD69 might be associated with the progression of *Mtb* infection to disease. The potential of CD69 as an indicator to predict infection reactivation, to decrease immune protection, and to differentiate between CCs with LTBI and ATB remains to be further defined.

## Data Availability Statement

All datasets presented in this study are included in the article/Supplementary Material.

## Ethics Statement

The studies involving human participants were reviewed and approved by the Ethical Committee of Shanghai Pulmonary Hospital (approval number K18-215Z), and informed consent was obtained from all subjects. The patients/participants provided their written informed consent to participate in this study.

## Author Contributions

X-YF, WS, and ZH conceived and designed this study. LW, Z-YC, LG, and RQ collected clinical samples and performed experiments. ZH performed statistical analyses. X-YF, ZH, and Z-YC interpreted the pooled results. ZH and Z-YC drafted the manuscript. X-YF and DL revised the manuscript. All authors approved the final manuscript.

## Conflict of Interest

The authors declare that the research was conducted in the absence of any commercial or financial relationships that could be construed as a potential conflict of interest.

## Supplementary Material

FIGURE S1 Flow cytometry gating strategy. Representative flow cytometric dot plots are shown. Briefly, CD4^+^ T cells were gated as CD3^+^ CD4^+^ cells and CD8^+^ T cells were defined as CD3^+^ CD4^–^ cells.

FIGURE S2 The MFI values of CD69 on CD4 T cells are shown.
