# Supplementary figures and images for: Decreased Expression of CD69 on T Cells in Tuberculosis Infection Resisters
Source: Front Microbiol. 2020 Aug 7;11:1901. doi: 10.3389/fmicb.2020.01901 (PMC7426741; doi:10.3389/fmicb.2020.01901)

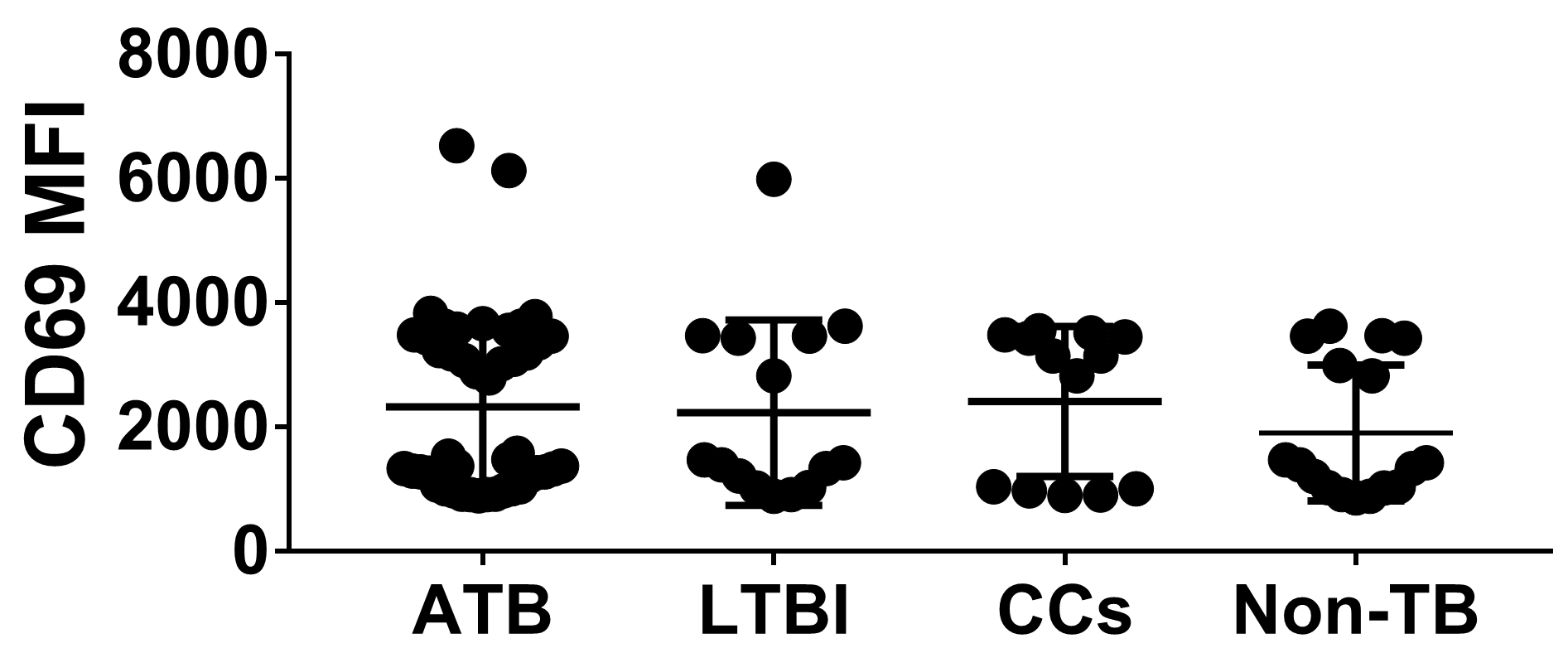

Supplement: FIGURE S2 — The MFI values of CD69 on CD4 T cells are shown. [file Image_2.TIF]
